# Glucose-6-phosphate dehydrogenase (G6PD) deficiency in girls: a diagnosis not to be missed (a case report)

**DOI:** 10.11604/pamj.2022.42.240.36270

**Published:** 2022-07-28

**Authors:** Yousra El Boussaadni, Abdelhakim Aboulfouyoul, Kaoutar Khabbache, Hanan Khalki, Abdallah Oulmaati

**Affiliations:** 1 Pediatric Department, *Centre Hospitalier Universitaire* Tanger Tétouan Al Hoceima (CHU TTA), Faculty of Medicine and Pharmacy of Tangier, Abdelmalek Essaadi University, Tangier, Morocco,; 2 Biochemistry Department, *Centre Hospitalier Universitaire* Tanger Tétouan Al Hoceima (CHU TTA), Faculty of Medicine and Pharmacy of Tangier, Abdelmalek Essaadi University, Tangier, Morocco

**Keywords:** Glucose-6-phosphate dehydrogenase (G6PD), child girl, hemolysis, case report

## Abstract

Glucose-6-phosphate dehydrogenase (G6PD) is a polymorphic enzyme encoded by the X chromosome. It protects the cell against hydrogen peroxide-induced damage and ensures an oxidative balance profile within the cell. The disease is more frequent in males, and rare cases are described in girls. We report an observation of a 7-month-old Moroccan girl hospitalized for acute hemolysis after consuming fava beans. The diagnosis of a G6PD deficiency was retained after an assay of the enzymatic activity that returned collapsed. After initial conditioning, a transfusion of phenotyped retinal ganglion cells (RGCs) is performed. The rapid evolution is favorable, and the child is discharged after therapeutic education sessions for the parents on the products to be avoided. Through this observation, we insist on the importance of neonatal screening in regions with a high prevalence of hemolysis in order to avoid diagnostic delays and also to prioritize the evaluation to be requested in an acute hemolysis state, to propose an education articulated around a preventive approach in children with this disease.

## Introduction

Glucose 6 phosphate dehydrogenase (G6PD) is a ubiquitous enzyme that is present in all cells. It catalyzes the first step of the pentose pathway, an alternative pathway to the Krebs cycle, to modulate the response to oxidative stress by forming Nicotinamide Adenine Dinucleotide Phosphate (NADPH). The gene coding for G6PD is located on the X chromosome; therefore, transmission is hereditary and sex-linked. The disease is more frequent in males, and rare cases are described in girls [1]. We report the observation of a girl hospitalized for severe acute hemolysis whose parents were free of the disease and whose diagnosis of a G6PD deficiency was made based on clinical and biological criteria.

## Patient and observation

**Patient information:** it is a female infant, 7-months-old, without any personnel history. Pregnancy and birth history were unremarkable, no parental consanguinity. The girl had a family history of hospitalizations of paternal cousins for generalized jaundice after ingestion of fava beans without authentic documents.

**Clinical findings:** the girl is admitted for jaundice, dark urine occurred 12 hours after ingestion of fava beans, in whom the clinical examination at admission, finds an obnubilated infant, hypotonic, icteric on a pale background, hemodynamically unstable (recoloration time extended to 5 seconds, tachycardia at 180 beats/min) and respiratory (polypneic at 65 cycles/min, saturation at 82% in ambient air) all evolving in a context of apyrexia quantified at 37.5°C, without hepato nor splenomegaly, the weight and height of the infant are at average.

**Time line and diagnostic assessment:** at this stage, several diagnoses were evoked, and a hemolysis assessment was performed, which came back in favor of a profound normocytic normochromic anemia (hemoglobin level at 2.3g/dl, mean corpuscular volume at 79 fl, mean corpuscular hemoglobin concentration at 32%) with a correct platelet count, a reticulocyte count of 98000/mm^3^, a collapsed haptoglobin <0.1 g/l, a predominantly free hyperbilirubinemia at 45mg/l, the complement of the etiological workup came back in favor of a negative Coombs test, a normal renal function. A standard hemoglobin electrophoresis profile and an enzyme assay are requested at this stage.

**Therapeutic intervention:** after initial conditioning, a transfusion of phenotyped RGCs is performed over 3 hours in an amount calculated according to the weight and the hemoglobin level, in view of the depth of the anemia and the absence of irregular agglutins.

**Follow-up and outcomes:** the rapid evolution is favorable, and the child is discharged after therapeutic education sessions for the parents on the products to be avoided, in particular medicines and certain foods commonly used in our culture, and also on the fact of consulting in case of appearance of signs of hemolysis. The infant is monitored in a pediatric hematology consultation with a 6-month follow-up. After discharge, the G6PD assay came back collapsed at: 1.2 IU/l g Hb in favor of a G6PD deficiency in the infant, and the check-up was completed with an assay in the parents.

**Informed consent:** parents of patient give informed consent.

## Discussion

Glucose 6 phosphate dehydrogenase catalyzes the first step of the pentose pathway, which is an alternative pathway to the Krebs cycle. This enzyme converts 6-glucose phosphate to 6-phosphogluconolactone, which is classically hydrolyzed to 6-phosphogluconate. During this reaction, a molecule of NADP+ is reduced to NADPH+ H+ [2,3]. Glucose 6 phosphate dehydrogenase deficiency hemolytic anemia is the most common erythrocyte enzymopathy in the world, with approximately 400 million affected individuals. A high deficiency prevalence is noted around the Mediterranean, sub-Saharan Africa, America and Southeast Asia [4,5].

According to the enzymatic activity (physio-chemical and chromatographic) of G6PD and taking into account the different clinical features, the World Health Organization (WHO) has defined 5 classes of variants of this enzyme, among which we note the “Mediterranean” type, variant A which represents 20% of the black African population, and also variant B which represents [6]. In most cases, the deficiency affects, in particular, the enzymatic stability involving amino acid substitutions, which consequently reduces the half-life of the protein by up to 8 days for the so-called most severe Mediterranean variant. It is, in fact, the reduced half-life that may be responsible for the clinical manifestations [1,6].

In the physiological state, during oxidative stress, hydrogen peroxide (H_2_O_2_) is produced in abundance and is toxic to the cell. This molecule is detoxified into 2 molecules of H_2_O by the reduced glutathione, while the glutathione oxidizes by losing its hydrogen to become oxidized glutathione again; this reaction takes place in parallel with the oxidation/reduction reaction of the NADPH/NADP couple using the cellular G6PD, which is only active at 2% in the normal state and only in the event of stress that this one is uninhibited by the drop in the NADPH in order to adapt to such aggression later [7].

During oxidative stress in G6PD-deficient patients, there is an accumulation of H_2_O_2_in the red blood cell due to the reduced half-life of G6PD and, consequently, an alteration of its membrane, which will induce precipitation of hemoglobin into Heinz bodies and a rupture of the red blood cell [1]. On the pathophysiological level, many agents are responsible for oxidative stress, in particular the ingestion of fava beans, as in the case of our patient, given the common consumption of fava beans in our context, followed by the intake of certain drugs such as synthetic antimalarials (chloroquine) as well as other infectious agents (*Escherichia coli*, beta-hemolytic streptococcus, and rickettsiae) or certain foods. These factors have the common characteristic of having redox properties [6,7]. Molecularly, the gene coding for this enzyme is located on the long arm of the X chromosome, which implies that transmission is hereditary and X-linked. Theoretically, boys carrying a mutated gene always have a deficit with variable clinical expression, whereas females may have a normal homozygous status or a homozygous or heterozygous deficit status. This last figure case is particular because they are patients carrying a deficient gene and a normal gene. According to the concept of lyonization (inactivation of the X or Lyon theory), each cell expresses only one X chromosome, which implies the presence of two cell populations in this category of patients: one expressing a normal G6PD gene and the other one a deficient G6PD gene with a ratio of 50/50 inconstant, resulting in a phenotypic variability between (standard, intermediate or deficient) [8].

Girls carrying the deficiency are scarce, and only a few cases are described in the literature [9]; they come from a couple where the father is affected, and the mother transmits, which is probably the case of our patient. The clinical presentation of a G6PD deficiency is typically that of acute hemolysis with abdominal pain associated with jaundice and dark urine related to hemoglobinuria, and this triad occurs on average 3 days after exposure to the oxidative agent; this presentation is variable depending on the age of the patient and also depending on the variant class. In the same context, the most severe forms are reported in patients with the Mediterranean variant leading to an intra- and extravascular hemolytic event often requiring transfusion with recourse to extra-renal purification in extreme cases [10]. In addition to the so-called typical form of acute hemolysis, other symptomatic forms have been described in the literature. The neonatal form, consisting of neonatal jaundice and early hyperbilirubinemia, has been described by some authors and may lead to a picture of kernicterus, which should lead to the diagnosis being evoked in the presence of such patterns and to the proposal of screening tests in high-risk populations [8]. Chronic hemolysis has also been reported either isolated or intermittently with acute attacks. These acute or intermittent hemolysis attacks can be triggered by the oxidizing agents mentioned above. Favism diagnosis necessarily implies a therapeutic education program for patients and their families with the provision of a list of drugs and foods adapted to our Moroccan context (Table 1) [11].

**Table 1:** G6PD deficiency; hemolytic foods and drugs

Food not allowed	
Food	Fava beans: beans: green, white, red, black Lentils; pea; chick peas; cinnamon; mushrooms; prickly pear
Drinks	Drinks containing quinine; verbena
Medicine and drugs	
To avoid	Nalidixic acid, sulfonamides and sulfones (dapsone, furosemid, sulfacetamide, sulfamethoxazole, sulfanilamide, Sulfasalazine, sulfisoxazole) antimalarials (quinine, chloroquine, mefloquine), nitrofurans, quinolones and fluoroquinolones (injectable and oral routes), rasburicase, phytomenadione (vitamin K), spiramycin (injectable and oral routes)
Not recommended at high posology	Acetylsalicylic acid, paracetamol, ascorbic acid
To be used under supervision	Methylene blue (oral and ophthalmic routes), nitric oxide, ciprofloxacin (ophthalmic and auricular routes), colchicine, doxorubicin, levodopa, isoniazid

The positive diagnosis is made after orientation and then a certainty biological assessment. The standard diagnostic evaluation for any situation of acute hemolysis is a blood count, which typically shows severe normocytic anemia with high reticulocytosis. This reticulocytosis may be minimal in the case of hemolysis of infectious origin. Hyperbilirubinemia with predominantly free bilirubin is also noted in G6PD hemolytic anemias with collapsed haptoglobin [12]. Being common in hemolytic states the referral assessment must be followed by a confirmatory evaluation of which the G6PD assay is a part. This assay is especially requested in male subjects of Mediterranean, African or Asian origin. However, deficiencies have been described in almost all populations of the world due to the migration of human beings. This confirmatory test consists of a study of the activity of the enzyme in vitro by respecting the conditions of the 5ml sample in an ethylenediaminetetraacetic acid (EDTA) bottle at a distance from any transfusion and, if possible, at a distance from the regenerative period because the deficit is predominant in the older red blood cells. The result may be falsely normal due to an excessive presence of young red blood cells (reticulocytes). The spectrophotometric assay of enzymatic activity is the quantitative reference test. It determines the degree of deficiency as severe or moderate according to the percentage of enzymatic activity [13].

In parallel, so-called screening tests should be proposed for newborns in high-risk populations, particularly the “fluorescent spot test” integrated into the guthrie, which can distinguish deficient newborns with an activity <10% from other normal subjects. With its low cost and time to perform, it remains the test of choice in populations with a high prevalence of this deficiency type, particularly in Africa. Thanks to this screening, progress in early diagnosis have been made in countries with a high prevalence of enzymopathy [14-16]. Other tests may be necessary, particularly in heterozygous girls, as biochemical tests cannot distinguish between the 3 subpopulations mentioned above. In this case, when there is a discrepancy between a suggestive clinical picture and a standard quantitative spectrometric G6PD assay, molecular biology by gene amplification (PCR) remains the method of choice. It allows direct sequencing of the deoxyribonucleic acid (DNA) of G6PD-deficient subjects and the characterization of an increasing number of new mutants. However, this method remains expensive and is not routinely available. In the case of solid reticulocytosis, the biochemical assay may be falsely normal, hence the interest in repeating it at a distance from the hemolytic episode [17,18].

Therapeutically, the treatment is mainly preventive in children with G6PD deficiency, including the avoidance of oxidizing agents (drugs, food and also toxins), through an integrated therapeutic education of the patient and his entourage (parents, family doctor), through a personalized card to the patient and a list of drugs to be taken and not to be taken in order to avoid acute hemolysis attacks. In parallel with therapeutic education, neonatal screening in at-risk populations (Black, Mediterranean, Asian) remains promising [16]. After the occurrence of hemolysis, the treatment remains symptomatic with frequent recourse to transfusion of red blood cells, especially in cases of favism. Still, transfusion is not the rule for all patients. This treatment can be supplemented by folic acid supplementation, which is hardly systematic, or by vitamin E, suggested by several authors as an anti-oxidant that can protect against chronic hemolysis in some forms of G6PD deficiency [19]. What is already known in this topic: the G6PD deficiency is the most common erythrocyte enzymopathy in the world, with approximately 400 million affected individuals. The gene coding for this enzyme is located on the X chromosome, which implies that transmission is hereditary and X-linked. The disease is more frequent in males, and rare cases are described in girls. What this study adds: we report an observation of a 7-month-old Moroccan girl hospitalized for acute hemolysis after consuming fava beans. Through this observation, we insist on the importance of neonatal screening in regions with a high prevalence of hemolysis in order to avoid diagnostic delays and also to prioritize the evaluation to be requested in an acute hemolysis state, to propose an education articulated around a preventive approach in children with this disease.

## Conclusion

G6PD deficiency may be asymptomatic in some patients and is triggered only after exposure to specific oxidizing agents, hence the interest in thinking about it when an acute hemolysis attack is triggered within 24 to 48 hours after exposure to an oxidizing agent in particular fava beans and is biologically confirmed by measuring erythrocyte G6PD enzymatic activity. The originality of our observation is to think of this pathology in both sexes in populations with a high prevalence, such as the Mediterranean population, when faced with hemolysis tables unexplained by the patient's context.

## References

[1] Cappellini MD, Fiorelli G (2008). Glucose-6-phosphate dehydrogenase deficiency. Lancet.

[2] Notaro R, Afolayan A, Luzzatto L (2000). Human mutations in glucose-6-phosphate dehydro genase reflect evolutionary history. FASEB J.

[3] Rossi R, Milzani A, Dalle-Donne I, Giustarini D, Lusini L, Colombo R (2002). Blood glutathione disulfide: in vivo factor or in vitro artifact. Clin Chem.

[4] Aubry P (2014). Déficits enzymatiques héréditaires du globule rouge (Enzymopathies héréditaires). Méd Trop.

[5] Mégarbane B (2008). Déficit en glucose-6-phosphate déshydrogénase: quand y penser et quelles précautions prendre. Réanimation.

[6] Wajcman H, Galacteros F (2004). Le déficit en glucose-6-phosphate déshydrogénase: protection contre le paludisme et risqué d´accidents hémolytiques. CR Biol.

[7] Beutler E, Gaetani G, Kaloustian V, Luzzatto L, Niwa S, Pannich V (1989). Glucose-6-phosphate dehydrogenase deficiency. Bulletin of the World Health Organization.

[8] Renault A, Mitanchez D, Corteya A (2017). Déficit en G6PD chez la fille à révélation néonatale. Revue de 4 cas cliniques. Arch Pediatr.

[9] Renault A, Mitanchez D, Cortey A (2015). Déficit en G6PD chez la fille: Qu´en est-il. Archives de Pédiatrie.

[10] Organisation Mondiale de la Santé (1990). Déficit en glucose-6-phosphate déshydrogénase. Bulletin de l´Organisation mondiale de la Santé.

[11] M Yamoul M (2016). Anémie hémolytique par déficit en G6PD chez l'enfant à propos de 30 cas.

[12] Dorche C (2000). Pathologie des enzymes de la glycolyse érythrocytaire. Revue Française des Laboratoires.

[13] Mura M, Saidi R, Wolf A, Moalic JL, Oliver M (2009). Anémie hémolytique congénitale par déficit en glucose-6-phosphate déshydrogénase. Med Trop (Mars).

[14] Kaddari F, Sawadogo M, Sancho J, Lelong M, Jaby D, Paulin C (2004). Dépistage néonatal du déficit en glucose-6-phosphate déshydro génase sur sang de cordon, centre hospitalier Delafontaine. Ann Biol Clin (Paris).

[15] Badens C, Leclaire M, Collomb J, Auquier P, Soyer P, Michel G (2001). Déficit en G6PD et ictère neonatal. Presse Med.

[16] Monchy D, Babin FX, Srey CT, Ing PN, von Xylander S, Ly V (2004). Déficit en G6PD, fréquence dans un groupe d'enfants d'âge préscolaire d'une région centrale du Cambodge. Med Trop (Mars).

[17] Coulibaly FH, Koffi G, Toure HA, Bouanga JC, Allangba O, Tolo A (2000). Galacteros, molecular genetics of glucose-6-phosphate dehydro genase deficiency in a population of newborns from Ivory Coast. Clin Biochem.

[18] Kwok CJ, Martin AC, Au SW, Lam VM (2002). G6PDdb, an integrated database of glucose-6-phosphate dehydrogenase (G6PD) mutations. Hum Mutat.

[19] Agence Nationale De Sécurité Du Médicaments Et Des Produits De Santé (2014). Médicaments et déficit en Glucose-6-Phosphate Déshydro génase (G6PD): classement des médicaments par substance active. Référentiel.

